# Endocytic signaling in leaves and roots: same rules different players

**DOI:** 10.3389/fpls.2012.00219

**Published:** 2012-10-02

**Authors:** Christian Craddock, Zhenbiao Yang

**Affiliations:** Center for Plant Cell Biology, Department of Botany and Plant Sciences, University of California, RiversideCA, USA

**Keywords:** auxin, ABP1, ROP RIC actin, endocytosis clathrin, microtubules

## Abstract

To take up proteins and other components required by the cell, cells internalize a portion of the plasma membrane (PM), which invaginates to form a closed vesicle within the cytoplasm in a process known as endocytosis. The major plant endocytic mechanism is mediated by clathrin, a protein that is necessary to generate a coated vesicle on the inner side of the PM. These vesicles bud away from the membrane generating a vesicle whose contents originated from outside of the cell and they can selectively concentrate or exclude compounds. The process is therefore of key importance to plant growth, development, signaling, polarity, and nutrient delivery. Rho family small GTPases are conserved molecular switches that function in many signaling events. Plants possess only a single Rho-like GTPase (ROP) family. ROPs are known to be involved in the control of cell polarity by regulating endocytosis. To contend with the high levels of regulation required for such processes, plants have evolved specific regulators, including the Rop-interactive CRIB motif-containing protein (RIC) effectors. Recent findings have demonstrated that ROP dynamics and the cytoskeleton (including actin microfilaments and microtubules) are interwoven. In this review, we summarize the current understanding of endocytosis in plants, with particular regard to the signaling pathways.

## INTRODUCTION

The generation of planar cell polarity (PCP) is a process involving the distribution of cellular structures or molecules asymmetrically. PCP establishment requires a mechanism for the formation of both intra-cell polarity and inter-cell polarity.

Rho-like GTPases (ROPs) from plants are the sole signaling small GTPases in plants and it is therefore expected that they have a role in numerous signaling events. ROPs are already known to participate in signaling pathways that regulate cytoskeletal organization and vesicular trafficking, and as a consequence have an impact on cell polarization, polar growth, and cell morphogenesis. Microtubules (MTs) and actin microfilaments (F-actin) are the two major cytoskeletal elements that play a key role in many cellular processes, including cell polarity and endocytosis.

In plants, the phytohormone auxin has a cardinal role in the coordination of many physiological functions, including growth and the development of cells and organs (Benkova et al., 2003; Friml et al., 2003; Blilou et al., 2005; Vieten et al., 2005; Weijers et al., 2005; Scarpella et al., 2006; Wisniewska et al., 2006; Grieneisen et al., 2007; Gao et al., 2008; Yang, 2008). To function, auxin must be dynamic both spatially and temporally (Santner and Estelle, 2009; Vanneste and Friml, 2009). In multicellular plants, this process is in part mediated by the polar distribution of the auxin efflux carriers PIN-FORMED (PIN) proteins, which are required for polar auxin transport and the formation of auxin gradients.

Asymmetric endocytosis and the recycling of PINs localized at the plasma membrane (PM) contribute to the polar localization of PINs (Geldner et al., 2003; Dhonukshe et al., 2008). More recently, auxin has been implicated as a self-organizing signal that causes the polarization of PIN proteins. The auxin signal that appears to regulate downstream ROPs involved in PCP is mediated through auxin binding protein 1 (ABP1). ABP1 has been proposed to regulate clathrin-mediated endocytosis in roots, and the ROP-dependent pavement cell (PC) interdigitation in leaves (Robert et al., 2010; Xu et al., 2010, 2011).

The signaling mechanisms involved in the formation of cell polarity, including ROPs, their close relationship with the cytoskeleton and endocytic trafficking are the focus of this review. The above-mentioned mechanisms are all conserved in plants and animals, and consequently advances in knowledge in the plant system may synergize advances in understanding similar mechanisms and processes in mammalian systems.

## SIGNALING AND ENDOCYTOSIS IN PAVEMENT CELLS

The formation of the jigsaw puzzle like shape of *Arabidopsis* leaf PCs epitomizes a long-standing question in cell and developmental biology. How does a field of cells precisely coordinate uniform cell polarity? Importantly, the interdigitation of PCs provides an excellent system for the investigation because interdigitation is a non-essential process. It is therefore possible to study the signaling mechanism with the use of overexpressing or knockout plant lines.

**FIGURE 1:**
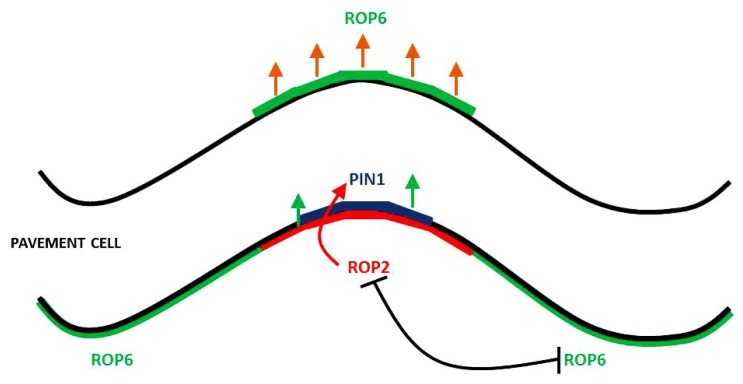
**Rho-like GTPase activation regions and PIN1 localization in leaf PCs.**ROP2 promotes the localization of PIN1 to the lobe tip which initiates a positive feedback loop in this region. The mutual exclusivity of ROP2 and ROP6 help restrict PIN1 to the lobe region.

In leaf PCs, the auxin cell surface receptor ABP1 mediates auxin signaling to coordinately activate two mutually exclusive ROP signaling pathways. They are activated in complementary lobe and indent regions on adjacent sides of the cell (**Figure 1**). A lobe in a cell corresponds to an indent in the adjacent cell. ROP2 and ROP4 promote lobe formation and are functionally redundant; ROP2 is the dominant ROP in lobe promotion and it is common to refer to ROP2 and ROP4 simply as ROP2. ROP6 is responsible for the promotion of indentations (Fu et al., 2002, 2005). Both ROP2 and ROP6 localize to and are activated at the PM (Xu et al., 2010, 2011). The localization of the auxin efflux carrier PIN1 to the lobe tips requires localized ROP2, indicating the existence of a localized auxin–ROP2–PIN1–auxin positive feedback loop that could be responsible for the generation and maintenance of localized auxin levels (Xu et al., 2010). However, it remains to be established how auxin-activated ROP2 regulates PIN1 polarization. ROP2 regulates the formation of multipolarity through its activation of RIC4 (Fu et al., 2005), a member of the ROP interacting CRIB motif-containing (RIC) family of ROP effector proteins (Wu et al., 2001). RIC4 induces the formation of cortical F-actin in the tips of PCs (Fu et al., 2005).

In the indenting zone, ROP6 activates RIC1, leading to the formation of well-ordered MT arrays, which promote indentation and inhibit ROP2 activation (Fu et al., 2005, 2009).

**FIGURE 2:**
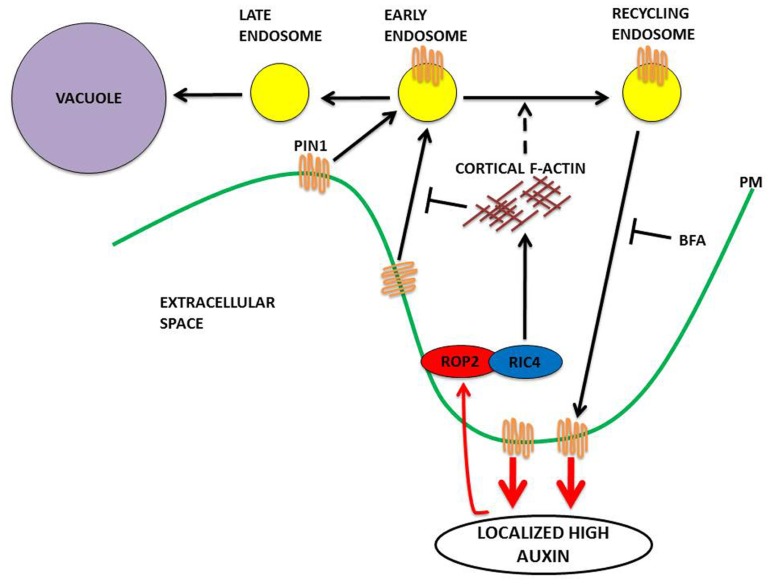
**A model in PCs for PIN1 polarization to the lobe regions of PCs via a ROP signaling mechanism.**ROP2 is activated by extracellular auxin in the lobe region. The activated ROP2–RIC4 pathway leads to the inhibition of PIN1 internalization through RIC4-dependent cortical F-actin, leading to PIN1 polarization at the lobe. The PIN1-based export of auxin leads to further ROP2 activation for completion of this feed-forward cycle. Recent data (Nagawa et al., 2012) indicates that the activated ROP2–RIC4 pathway has a role in the promotion of endosomal trafficking from early endosomes to recycling endosomes (Brefeldin A inhibits ADP ribosylation factor GEF and prevents endosomal recycling, the accumulation of internalized PIN1 in aggregates known as BFA bodies in plant cells). Endocytosed material can then be recycled back to the PM. Material maintained in early endosomes as they mature becomes internalized in late endosomes. The multivesicular structure of the late endosomes allows membrane fusion with the vacuole. Proteins within the late endosomes are delivered to the vacuole for degradation.

ROP2 inactivates RIC1, which causes the suppression of well-ordered cortical MTs, thus preventing outgrowth as MTs are excluded from outgrowing lobe tips (Fu et al., 2002, 2005). With the local activation of ROP2–PIN1 in the lobe region, ROP6 is suppressed at this site, given that the ROP2 and ROP6 pathways are mutually exclusive (Fu et al., 2009). Within an indent region, ROP6 activates RIC1, which leads to the creation of highly ordered MT arrays, which promote further indentation and inhibit ROP2 activity (Fu et al., 2005, 2009). It is hypothesized that the mutual inhibition between the ROP2 and ROP6 pathways transforms the initial uniform pool of auxin into localized extracellular auxin pools that enable the sustainment of ROP2 and ROP6 activity on opposing sides of the extracellular pool of auxin. It is thought that this interdigitated patterning of ROP2 and ROP6 activation could give rise to the lobe and indentation patterning that is observed between neighboring cells. It was recently shown that the rapid activation of the antagonizing ROP2 and ROP6 pathways require ABP1-dependent auxin perception (Xu et al., 2010). This work demonstrated that exogenous auxin promotes interdigitation in PCs, whereas a reduction in endogenous auxin suppresses interdigitation (Xu et al., 2010). Auxin inhibits PIN internalization (Paciorek et al., 2005; Dhonukshe et al., 2008), and consistent with this, the localization of PIN1 to the lobe tips (Fu et al., 2005) was found to be dependent on ROP2, which is activated in the same PM region where PIN1 is located (Xu et al., 2010). The evidence is suggestive that PIN1-directed auxin efflux is involved in the positive feedback regulation of ROP2 (Fu et al., 2002, 2005). This model is consistent with that of roots and guard cells, where constitutive activation of ROPs inhibited the internalization of the endocytosis marker FM-64 (Bloch et al., 2005; Sorek et al., 2010; Hwang et al., 2011). ROP2 was found to inhibit PIN1 endocytosis in the lobe regions of PCs. A series of elegant studies using the Dendra2 photo-convertible fluorescent protein revealed that in wild type, PIN1 endocytosis was found to occur in the indentation regions but not in the lobe regions. In contrast, expression of dominant negative ROP2 induced PIN1 endocytosis in the lobe region. Following the transient expression of PIN1–GFP in *rop2* mutant PCs, revealed that ROP2 is required for the inhibitory effect of auxin on PIN1 endocytosis. A final experiment using *ric4* mutant knock down and PIN1–GFP demonstrated that RIC4 has a role in promoting the accumulation of cortical F-actin in the lobe region and in turn inhibiting PIN1 endocytosis through RIC4-dependent F-actin. Recent data, summarized in **Figure 2**, show that the ROP2/RIC4 pathway inhibits clathrin-dependent PIN1 endocytosis thereby leading to PIN1 polarization. The direction of auxin movement is dependent on PIN auxin transporters, which constantly undertake endocytic recycling (Dhonukshe et al., 2007, 2008; Kleine-Vehn et al., 2011), with the polar location of PIN at the PM determining the direction of auxin flow between cells (Petrasek et al., 2006).

Although the nature and morphology of the recycling endosome remains elusive, plant early endosomes (EEs) and late endosomes (LEs) have been shown to correspond to the *trans*-Golgi network (TGN) and multivesicular bodies (MVBs), respectively. Endosomes are cellular organelles that appear to be involved in both the endocytic and biosynthetic pathways in plants (De Matteis and Luini, 2008; Foresti and Denecke, 2008). In the endocytic pathway, EEs receive internalized material from the PM and either recycle it back to the cell surface or target it for degradation, thereby acting as an important protein sorting station in the endocytic pathway, which is fundamental to ensure establishment and maintenance of cell polarity and homeostasis (Geldner, 2004; Geldner et al., 2004; Lam et al., 2007b; Otegui and Spitzer, 2008). In addition, EEs play a role in the biosynthetic pathway as they can receive newly synthesized material from the TGN and either sort it to the endosome/lysosome or recycle it back to the PM via the recycling endosome (De Matteis and Luini, 2008).

Studies using the endocytic tracer FM4-64, indicate that the VHA1-labeled TGN is an EE given that it displays detectable steady state levels of FM4-64 prior to the labeling of the PVC (Dettmer et al., 2006; Lam et al., 2007a). These studies in conjunction with morphological observations that both the TGN and the EE can present clathrin budding profiles (Payne and Schekman, 1985; Hillmer et al., 1988; Pearse and Robinson, 1990) further support the TGN and EEs being the same compartment (Geldner, 2004; Lam et al., 2007a).

Proteins within the EEs that are destined for degradation are sorted into another subdomain, which will form MVB/LEs (Murk et al., 2002). MVBs/LEs mediate the delivery of vacuolar-destined proteins to the vacuole through fusion with the tonoplast. It was shown that LE/MVBs contain visibly distinct morphological regions together with membrane domains enriched in two GTPases, Rab7, and Rab9, that regulate late endocytic traffic and LE/MVB to EE/TGN recycling, respectively (Gruenberg, 2001; Barbero et al., 2002), inferring that LEs/MVBs derive from TGN/EEs. Earlier evidence indeed demonstrated that the formation of LEs/MVBs from EEs/TGN is induced by the ubiquitination of receptors, as illustrated by the epidermal growth factor (EGF; Hicke and Riezman, 1996). More recent evidence also points toward LEs/MVBs being derived through the maturation of EEs/TGN (Scheuring et al., 2011). Importantly, their experiments suggest that the inhibition of clathrin-mediated transport does not halt the transport of soluble cargo bearing vacuolar sorting determinants to the vacuole. Therefore the widely held concept of the anterograde trafficking of proteins occurring via the recognition of sorting signals and trafficking through vesicles moving between stable compartments is not supported by this evidence. Instead the evidence supports the idea that anterograde trafficking occurs in the absence of CCVs and the recycling of receptors (Scheuring et al., 2011).

## REGULATION OF ENDOCYTOSIS IN ROOTS

In roots, the mechanisms underlying apical and basal polarization appear similar to the coordination of polarity in leaves. In roots, recent findings have shown that a signal module composed of auxin, ABP1, ROP6/RIC1, clathrin, PIN1/PIN2 act as an integral component of the feedback regulation of auxin transport during root development.

Recent evidence indicates that ROP6 affects endocytosis and is involved in PIN internalization (Chen et al., 2012). Subsequent experiments revealed that the uptake of FM4-64 increased in the roots of *rop6* or *ric1* mutant plant lines, whereas uptake was reduced in the presence of constitutive* rop6* expression (Chen et al., 2012). In addition, visualization of clathrin heavy chain with the aforementioned plant lines revealed that ROP6 signaling negatively regulates clathrin-mediated endocytosis (Chen et al., 2012). *rop6* and *ric1* mutants displayed lower levels of sensitivity to auxin indicating the ROP6/RIC1 pathway is involved in and acts downstream of both auxin regulation and ABP1 signaling in the regulation of clathrin-mediated endocytosis in roots (Chen et al., 2012). The role of the ROP6/RIC1 pathway in endocytosis roots is similar to the regulation of PC interdigitation in leaves (Xu et al., 2010; Nagawa et al., 2012). Both pathways possess the auxin feedback module composed of auxin–ABP1–ROP–clathrin-mediated endocytosis-PIN1/PIN2 localization, although there are key differences in how the individual steps are performed. In PCs, auxin acts via ABP1 to activate the ROP2 pathway and inhibits clathrin-dependent endocytosis leading to PIN1 polarization at the lobe (Xu et al., 2010; Nagawa et al., 2012). In roots, ABP1 seems to act as a positive regulator of clathrin-mediated endocytosis, whereas auxin acts as the inhibitor (Chen et al., 2012). The relatively mild phenotype displayed in the analyzed ROP6 genotypes indicates functional redundancy with other ROPs such as ROP9 and ROP11 (Bloch et al., 2005). Very recent findings have begun to link auxin signaling to PIN-mediated pattern formation and morphogenesis in roots. A genetic screen found that the absence of SPIKE1 leads to increased lateral root density and retarded gravitropic responses matching the phenotype observed in *pin2* knockouts (Lin et al., 2012). Mutant *spk1* plants induced PIN2 internalization that could not be suppressed by auxin, equivalent to *rop6* and *ric1* mutants. Moreover, SPIKE1 was required for auxin induction of ROP6 activation.

**FIGURE 3:**
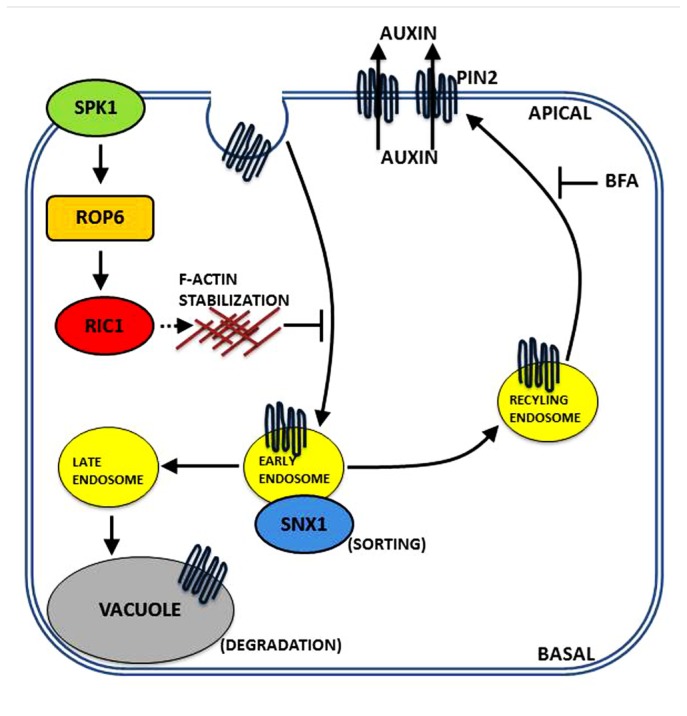
** A model in roots for PIN2 polar distribution via a ROP signaling mechanism.** Data indicate that the auxin-mediated inhibition of polar PIN2 internalization is regulated by the SPK1–ROP6–RIC1 pathway. Auxin is proposed to activate the SPK1–ROP6–RIC1 pathway and inhibit PIN2 internalization. The localized inhibition of PIN2 internalization via ROP6 signaling causes PIN2 to be retained in the PM, which generates a positive feedback mechanism for maintaining polar PIN2 distribution to the PM.

The current model for the polar distribution of PIN2 via the ROP-based signaling pathway is presented in **Figure 3**.

## CONCLUSION

Recent findings suggest that PIN internalization by ROP-based auxin signaling is a mechanism responsible for the regulation of polar auxin trafficking in plants. The ROP2/RIC4 pathway being responsible for the induction of F-actin in PCs which leads to the inhibition of PIN1 internalization necessary for PIN1 polarization to the lobe tips (Nagawa et al., 2012). The ROP6/RIC1 pathway functions in roots to inhibit PIN2 internalization through the stabilization of F-actin. A key distinction is that ABP1 activates the ROP pathway in PCs (Xu et al., 2010; Nagawa et al., 2012), whereas in roots ABP1 is responsible for inactivation of the ROP pathway (Chen et al., 2012). Future studies will hopefully elucidate whether this finding is due to differences in auxin concentration required to activate the ROP pathways in different tissues.

Whilst recent advances strongly suggest that the ROP-based auxin signaling that regulates PIN internalization is a widespread mechanism for the modulation of auxin transport in plants, key questions remain. Including for example the involvement of the ABP1 pathway in cytoskeletal dynamics and cell polarity. A resourceful use of biochemistry, forward and reverse genetics, and imaging are necessary to identify the remaining components to obtain a fuller understanding of the signaling events regulating endocytosis in plants.

## Conflict of Interest Statement

The authors declare that the research was conducted in the absence of any commercial or financial relationships that could be construed as a potential conflict of interest.
